# Interrelation between miRNAs Expression Associated with Redox State Fluctuations, Immune and Inflammatory Response Activation, and Neonatal Outcomes in Complicated Pregnancy, Accompanied by Placental Insufficiency

**DOI:** 10.3390/antiox12010006

**Published:** 2022-12-21

**Authors:** Vladislava A. Gusar, Angelika V. Timofeeva, Vitaliy V. Chagovets, Mikhail Yu. Vysokikh, Nataliya E. Kan, Ludmila A. Manukhova, Maria V. Marey, Gennadiy T. Sukhikh

**Affiliations:** 1 Laboratory of Applied Transcriptomics, Federal State Budget Institution “National Medical Research Center for Obstetrics, Gynecology and Perinatology Named after Academician V.I. Kulakov”, Ministry of Healthcare of the Russian Federation, Oparin Str. 4, Moscow 117997, Russia; 2 Laboratory of Metabolomics and Bioinformatics, Federal State Budget Institution “National Medical Research Center for Obstetrics, Gynecology and Perinatology Named after Academician V.I. Kulakov”, Ministry of Healthcare of the Russian Federation, Oparin Str. 4, Moscow 117997, Russia; 3 Laboratory of Mitochondrial Medicine, Federal State Budget Institution “National Medical Research Center for Obstetrics, Gynecology and Perinatology Named after Academician V.I. Kulakov”, Ministry of Healthcare of the Russian Federation, Oparin Str. 4, Moscow 117997, Russia; 4 A.N. Belozersky Research Institute of Physico-Chemical Biology, M. V. Lomonosov Moscow State University, Moscow 119992, Russia; 5 Federal State Budget Institution “National Medical Research Center for Obstetrics, Gynecology and Perinatology Named after Academician V.I. Kulakov”, Ministry of Healthcare of the Russian Federation, Oparin Str. 4, Moscow 117997, Russia

**Keywords:** microRNA/miRNA, newborn, perinatal outcomes, oxidative stress, immune response, inflammatory, placental dysfunction

## Abstract

Redox disbalance in placental cells leads to the hyperproduction of reactive oxygen species (ROS), it mediates the dysregulation of the maternal immune tolerance to a semi-allogenic fetus, inducing pro-inflammatory reactions, and it plays a central role in perinatal complications and neonatal disease programming. Microvesicles, which provide transplacental communication between a mother and fetus, contain microRNAs (miRNAs) that are sensitive to oxidative stress (OS) mediators and can control the balance of ROS production and utilization in target cells. In the context of this paradigm, we evaluated the markers of redox balance—MDA and 4-HNE for OS and GPx, and SOD, CAT, and GSH for the antioxidant system in the cord blood plasma of newborns diagnosed with fetal growth restriction (FGR)—by using polarography, spectrophotometry, and Western blotting. The expression of miRNAs associated with OS, immune and inflammatory responses in the blood plasma of newborns with intrauterine pneumonia (IP), neonatal sepsis (NS) and respiratory distress syndrome (RDS) was evaluated by a quantitative RT-PCR. Significant differences in the MDA level and reduced GPx and CAT activity were co-found for early-onset FGR (i.e., <34 gestational age). Significant correlations were found with a low birth weight by Apgar scores with reduced levels of antioxidant enzymes. Indeed, the level of OS markers increased in early-onset FGR in newborns with an extremely low body weight and high echogenicity of the periventricular zones, and reduced in late-onset FGR in newborns with IP, hyperbilirubinemia, intraventricular hemorrhage (IVH) and cerebral cysts. A prognostic model (AUC = 1; cutoff—0.5) was developed to assess the risk of IVH in newborns diagnosed with FGR based on the assessment of the OS markers (i.e., MDA + 4 HNE + CAT + GSH). A significant increase in the miR-127-3p expression was found in the plasma of newborns with NS (<32 GA; *p* ≤ 0.03 and >32 GA; *p* ≤ 0.009), IP (>32 GA; *p* ≤ 0.0001), and RDS (>32 GA; *p* ≤ 0.03). At the same time, the expression of miR-25-3p (*p* ≤ 0.03) was increased only in newborns with NS (>32 GA; *p* ≤ 0.03). The risk of developing IVH for premature newborns with IP (AUC = 0.8; cutoff—0.6) and NS (AUC = 0.68; cutoff—0.49) was assessed based on the miR-25-3p and miR-127-3p expression. Several key transcription factors were identified as the targets of studied miRNA since they are involved in the regulation of OS (NRF2), signaling and activation of the immune response (PRDM1, CCL26) and, also, inflammatory responses (NFKB1). The study of these miRNAs showed that they are involved in the modulation of processes leading to perinatal complications. Moreover, miR-127-3p is related to pro-inflammatory reactions and the formation of the macrophage phenotype in newborns with IP, NS, and RDS, while miR-25-3p is associated with an inhibition of macrophage migration and activation of antioxidant enzymes, which may prevent the development of oxidative damage in newborns with NS.

## 1. Introduction

Perinatal complications and pregnancy outcomes, as well as an unfavorable neonatal prognosis, are often associated with “major obstetric syndromes”, in particular preeclampsia (PE) and fetal growth restriction (FGR) [1,2]. In recent years, researchers from different countries have reached a consensus that the pathophysiology of these syndromes based on the dysfunction of the placenta, is associated with a decrease in the uteroplacental blood flow and subsequent ischemia/reperfusion injury of the uteroplacental complex. This ultimately leads to release into either the maternal circulation bloodstream of the mother and developing fetus of various inflammatory mediators, including pro-inflammatory cytokines, pro-oxidants and products of peroxidation after oxidative stress (OS) [3,4,5,6,7,8,9]. The main contributors of OS are reactive oxygen species (ROS) produced by various cell types. This definition covers a wide range of highly reactive oxygen-containing compounds, including free radicals (e.g., superoxide, hydroxyl and peroxyl radicals) and secondary products of radical reactions—non-radical compounds (e.g., hydrogen peroxide, singlet oxygen, peroxynitrite, lipid hydroperoxide, hypochlorite anion, and ozone) [10]. ROS are formed both as co-products of oxidative metabolic processes and in response to exogenous signals such as pathogens or endogenous signals (the activation of immune cells, inflammation, ischemia, infection, etc.) [11]. These compounds are important signaling molecules, acting as second messengers with participance in maintaining the physiological functions of cells—growth, proliferation and survival. It is important to note that low levels of ROS induce the activation of vital signaling pathways, while their high level promotes OS, which has a damaging effect on cells and tissues, leading to the development of various pathological conditions [10,12]. The balance of ROS is mediated by the functioning of the cellular antioxidant defense system, the key components of which are superoxide dismutase (SOD1, SOD2 and SOD3), catalase (CAT), glutathione peroxidase (GP1, 4) and the low molecular weight compound glutathione (GSH) [10,11]. Maintaining the intracellular and tissue balance of ROS is also regulated by small non-coding molecules called microRNAs (miRNAs). Changes in their expression can lead to a shift in this balance, causing a pathologically high level of oxidative damage in the placenta, its dysfunction, and the subsequent disturbance of fetal growth, development, and perinatal complications [13]. A number of studies have shown that hypoxia and OS affect miRNA secretion and their packaging into exosomes [14,15,16], causing an affected communication between the mother and fetus in placenta-associated diseases [17]. A growing body of research indicates that intracellular ROS can either inhibit or induce the expression of OS-sensitive miRNAs (the so-called “redoximiRs”), leading to various biological effects through the regulation of their target genes, in particular, Nrf2, SIRT1, NFκB, and the signaling pathways involved in OS [18,19]. In addition, ROS can modulate the biogenesis of some redoximiRs [20] involved in ROS-mediated signaling [21]. It is also significant that the expression and activity of key components of the antioxidant system are also regulated by certain miRNAs [20]. The close relationship of OS with the immune and inflammatory response is obvious: the production of ROS by cytokines induces inflammation, which in turn, enhances oxidative damage [12]. At the same time, miRNAs secreted by immune cells are able to mediate the M1 or M2 types of polarization of macrophage precursors [22].

The imbalance between an increased production of ROS and a decrease/absence of antioxidant protection is a significant factor in the pathogenesis of a number of neonatal diseases, especially for preterm infants. In particular, the manifestation of neonatal respiratory distress syndrome (RDS), broncho pulmonary dysplasia, retinopathy of premature, necrotizing enterocolitis, neonatal sepsis, brain damage such as periventricular leukomalacia, hypoxic-ischemic encephalopathy, and intraventricular hemorrhage (IVH) were observed [23,24,25,26,27,28,29]. Indeed, an increase has been previously shown in OS markers and a decrease in the antioxidant status in the blood of mothers with PE and in the cord blood of newborns, that correlates with premature, low birth weight, and neonatal diseases (e.g., FGR, RDS, and neonatal sepsis) [30]. The consequences of complications in infants born by mothers with PE also include a variety of clinical manifestations, such as thrombocytopenia, neutropenia, increased blood pressure, increased risk of stroke, adverse neurodevelopmental outcomes, and cognitive impairment [31].

Currently, there are practically no data on the role of OS-associated miRNAs in the regulation of the immune and inflammatory responses that serve as triggers for neonatal diseases caused by placental dysfunction [32]. In our previous study, we evaluated the redoximiRs expression (miR-125b-5p and miR-451a) in placenta and in cord blood of fetuses with FGR. It was found that the level of these miRNAs significantly changed in newborns with RDS and a very low birth weight (VLBW) and correlated with Doppler data (an increase in the resistance index in the umbilical artery and decrease in cerebral blood flow), maternal platelets and Apgar scores [33]. Based on these data, we suggested that a cross-talk violation of ROS and redoximiRs in complicated pregnancy may lead to the subsequent intrauterine clinical manifestation of various pathological states in newborns. In this regard, the aim of this work was to study the pro- and anti-oxidant markers of cord blood plasma of newborns with FGR, as well as to evaluate the miRNAs expression that regulates redox homeostasis, and immune and inflammatory responses, in an independent cohort of newborns with various adverse outcomes due to placental dysfunction. In addition, a search was carried out for correlations between changes in the level of redox markers, the miRNAs expression, and the clinical characteristics of newborns.

## 2. Materials and Methods

### 2.1. Study Design and Patient Cohort

**Figure 1:**
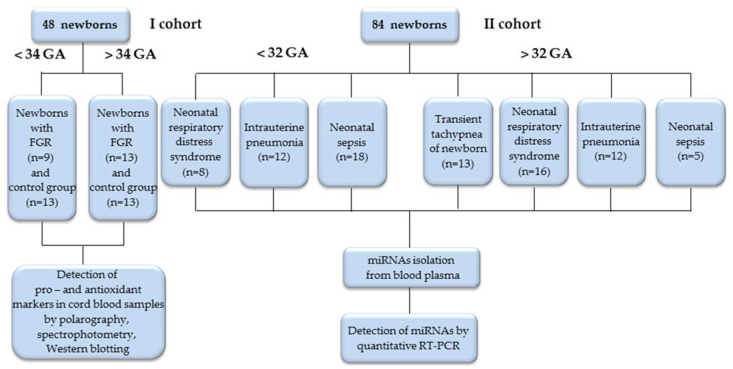
Flowchart of the study population.

This study included newborns with adverse outcomes due to placental dysfunction, born to mothers who were clinically observed at the V. I. Kulakov National Medical Research Center for Obstetrics, Perinatology and Gynecology, Ministry of Healthcare of the Russian Federation with diagnoses of PE, FGR, Gestational Diabetes Mellitus, gestational hypertension, thrombophilia and fetal acute hypoxia. The first cohort (I) included 48 newborns with early-onset (< 34 weeks; GA) and late-onset (> 34 weeks; GA) FGR according to the Delphi procedure [34], as well as newborns of a control group of the corresponding gestational age. Cohort I cord blood plasma samples were used to evaluate the markers of the pro- and anti-oxidant systems. In our previous study, the expression of OS-associated miRNAs was estimated in these samples [33]. Cohort II included 84 newborns, divided into two groups according to their gestational age (<32 and >32 weeks; GA), each of which included subgroups according to the diagnosis at birth: respiratory distress syndrome (RDS), intrauterine pneumonia (IP), neonatal sepsis (NS), and transient tachypnea (TT). Blood plasma samples from the newborns of Cohort II were taken to estimate the miRNAs expression associated with OS, and immune and inflammatory responses (Figure 1).

### 2.2. Clinical State Assessment of Newborns with Adverse Neonatal Outcomes

The clinical characteristics of newborns with FGR (Cohort I) are detailed in our previous study [33]. Newborns included in Cohort II, after primary stabilization of their state in the delivery room, were admitted to the neonatal intensive care unit (NICU). The blood samples were taken in the NICU in the first hours of a newborn’s life, and their subsequent examination was carried out in accordance with the protocol developed in the NICU of the V. I. Kulakov National Medical Research Center for Obstetrics, Perinatology and Gynecology, Ministry of Healthcare of the Russian Federation (i.e., the procedure for the examination of newborns suspected of infectious pathology and rules of antibacterial therapy, adopted at the Department of Resuscitation and Intensive Therapy for Newborns at the Kulakov Research Center for Obstetrics, Gynecology and Perinatology). 

The protocol for examining a newborn’s state included: radiologic investigation of the chest cavity, microbiological investigation, a blood panel (including the absolute number of leukocytes, platelets, neutrophils and the calculation of the neutrophil index (NI)), and the study of inflammatory proteins. To assess the severity of respiratory disorders, the scales of Silverman–Anderson [35] and Downes [36] were used. On the Silverman scale, a score of 0 points indicates the absence of disorders, from 1 to 3 points for the initial signs, and 4–5 points for the average severity of respiratory disorders. With a total score of 6 points or more in newborns, a syndrome of severe respiratory disorders is stated. On the Downes scale, a score of 2–3 points correspond to mild RDS, 4–6 points to moderate RDS, and more than 6 points to severe RDS. To assess the severity of the state and multisystem failure, the NEOMOD scale was used, which allows for assessing the state of the central nervous system, cardiovascular system, the respiratory organs, gastrointestinal tract, hemostasis system, urinary system, and the acid-base balance, regardless of the gestational age and birth weight (e.g., 0 points—no disorders, and 1 point—with moderate and 2 points—with severe dysfunction). With a scale value of 5 points or more, the risk of death is likely [37]. 

Based on the data examination at the age of 72 h of life, a conclusion was made about the presence or absence of an infectious disease in a newborn. When respiratory disorders appeared immediately after birth based on the background of the lack of a need for additional oxygen supply, as well as an increase in the bronchovascular pattern according to X-ray data, a diagnosis of TT was made. Severe respiratory disorders and dependence on additional oxygen supply, not associated with the development of an infectious process, served as the basis for the diagnosis of RDS. The presence of at least two clinical and one laboratory sign associated with the development of systemic inflammatory response syndrome (SIRS), as well as convincing signs of pneumonia according to X-ray data (i.e., the presence of focal and infiltrative shadows, indirect signs—an increased bronchovascular pattern, or hypopneumatization of lung tissue) served as the basis for the diagnosis of IP. The diagnosis of early-onset and late-onset NS was established based on the detection of a focal point of infection or a positive blood culture, the presence of signs of SIRS, at least one of which was hematological, and symptoms of multisystem failure. In accordance with the clinical guidelines developed in the NICU and the Surviving Sepsis Campaign International Guidelines [38], a differential diagnosis was made between IP, early-onset NS, RDS, and TT. A number of clinical characteristics of the newborns and maternal diagnoses are presented in Table 1. Newborns were also diagnosed with perinatal complications, including DIC, hyperbilirubinemia, congenital anemia, asphyxia, cerebral ischemia, seizures, gastrointestinal dyskinesia, gastric bleeding, and non-specific enterocolitis (presented in the supplementary Table S1).

### 2.3. Detection of Pro- and Anti-Oxidant Markers in Umbilical Cord Blood of Newborns with FGR

Samples of umbilical cord blood plasma taken during the caesarean section from the umbilical vessels (predominantly artery vessels and immediately after ligation closer to the placental end) of early- and late-onset FGR and the control groups of the corresponding gestational age. The plasma samples were used to study the functional state of the pro- and anti-oxidant system of the newborns with FGR based on the determination of lipid peroxidation products (malonic dialdehyde (MDA) and 4-Hydroxynonenal (4-HNE)), non-enzymatic (glutathione) and enzymatic antioxidant systems (catalase (CAT), superoxide dismutase (SOD) and glutathione peroxidase (GPx)). Using a Thermo Scientific Multiskan GO spectrophotometer, the optical density of the MDA adduct with thiobarbituric acid (λ = 535 nm) was determined according to [39], and the content of total glutathione (tGSH), reduced (GSH) and oxidized (GSSG) (λ = 412 nm) was determined as described in [40]. Using a double-beam spectrophotometer Cary Varian II, the optical density of 4-HNE (λ = 360–380 nm) was determined according to [41]. The SOD activity (λ = 347 nm) was determined by the inhibition of adrenochrome oxidation after hemoglobin extraction in a chloroform–ethanol–water mixture according to [42]. The GPx activity was determined at λ = 340 nm according to [43]. The CAT activity was measured by the polarographic method [44] on a Hansatech oxigraph by determining the amount of O_2_ that is formed in the decomposition of H_2_O_2_ with the participation of catalase. The content of SOD1 and SOD2 in the cord blood plasma was assessed by Western blotting using monoclonal antibodies (ab13498 and ab74231; Abcam, Cambridge, UK) by normalization of the signal to the housekeeping protein (b3-tubulin and ab18207; Abcam, USA). Three replicates per sample were used for every protein. The mean and standard deviation of these replicates were used for the analysis.

### 2.4. Blood Collection of Newborns, miRNAs Extraction from Blood Plasma and Real-Time Quantitative RT-PCR

The samples of blood plasma were collected into VACUETTE^®^ tubes containing EDTA (BectonDickinson Canada, Mississauga, ON, Canada). The following protocol was used: whole blood was centrifuged at 300× *g*, at 4 °C for 20 min, and then the supernatant was centrifuged at 16,000× *g* for 10 min. A total of 200 μL of the prepared plasma was used for the isolation of miRNAs by an miRNeasy Serum/Plasma kit (Qiagen, Hilden, Germany). An endogenous control (5.6 × 10^8^ copies of cel-miR-39 (miScript Primer Assay, Qiagen)) was added to a plasma sample for evaluation of the efficiency of isolation and subsequent cDNA synthesis for a quantitative RT-PCR. The extraction stages were carried out at an automatic station (QIAcube) in accordance with the protocols of the manufacturer, Qiagen. 

An miScript II RT Kit and miScript SYBR Green PCR Kit (Qiagen, Hilden, Germany) were used to evaluate the miRNA expression in the blood plasma samples from the newborns. The reaction was performed using a StepOnePlus device (Applied Biosystems, Foster City, CA, USA). The following RNA-specific sense primers were used: hsa-miR-25-3p MIMAT0000081 (5′-CATTGCACTTGTCTCGGTCTGA, Tm = 56 °C), hsa-miR-127-3p MIMAT0000446 (5′-TCGGATCCGTCTGAGCTTGGCT, Tm = 52 °C), and cel-miR-39 (Tm = 55 °C). The threshold level of expression was Ct ≤ 38. The stages were carried out in accordance with the protocols of the manufacturer, Qiagen.

### 2.5. Signaling Pathway Analysis

The analysis of the signaling pathways and interaction of the studied miRNAs with the relative target genes was assessed using the miRTarBase 9.0 database, available online: https://mirtarbase.cuhk.edu.cn/ (accessed on 19 December 2022) and GeneCards, available online: https://www.genecards.org/ (accessed on 19 December 2022). The miRTarBase database automatically generated miRNA interaction networks with target genes when searching for the targeted miRNAs in the current study. Then, only those miRNA-target pairs were selected. The potential miRNA-gene interaction was confirmed experimentally using evidence-based methods (particularly, a Reporter assay, Western blotting, and quantitative PCR) through the miRTarBase database results. Then, a search was performed for the participation of the selected target genes regulated by the studied miRNAs in the signaling pathways using GeneCards.

### 2.6. Statistical Analysis

The miRNAs expression in newborns less than 32 weeks of gestation was compared between the groups with RDS, IP, and NS, and in infants over 32 weeks of gestation, between the groups with RDS, IP, NS, and TT. The level of miRNA expression was determined by the 2^−ΔΔCT^ method [45], using a cel-miR-39 miScript Primer Assay (Qiagen) as an internal control. The statistical significance of the difference between the clinical parameters and the miRNA expression in the groups under study was assessed by the Wilcox–Mann–Whitney test using scripts written in the R language (https://www.R-project.org/, accessed on 19 December 2022). The Spearman nonparametric rank correlation method was used to evaluate the relationship between the studied miRNAs expression, clinical parameters, and proteins’ level. The normality of the clinical parameters distribution was evaluated by the Shapiro–Wilk test. A statistical analysis was performed using the Student’s test with a normal distribution of the parameter and using the Mann–Whitney test when the distribution did not correspond to the law of normal distribution. The Bonferroni correction was used for multiple comparisons. To describe the quantitative data having a normal distribution, the mean value (M) and standard deviation (SD) in the M ± SD format were used. In the case of a non-normal distribution, the parameter was described as the median (Me) and quartiles Q1, Q3 in the format Me (Q1–Q3). 

## 3. Results

### 3.1. Evaluation of Pro- and Anti-Oxidant Markers in Umbilical Cord Blood of Newborns with FGR 

**Figure 2:**
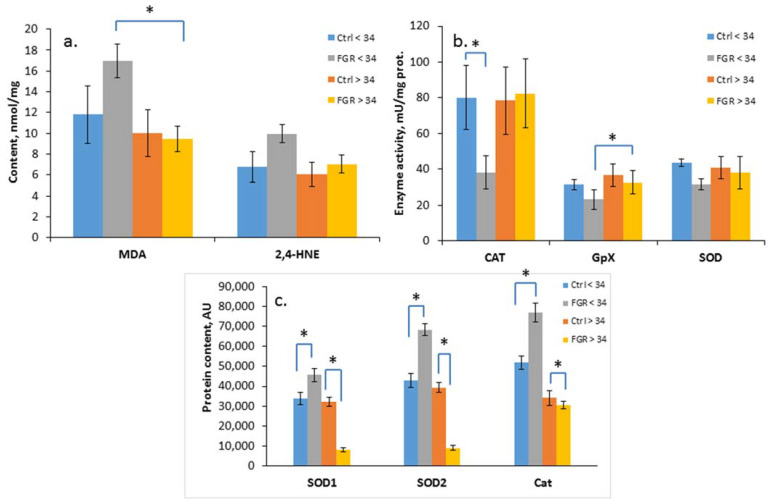
Content of MDA and 4-HNE (**a**); SOD, CAT and GPx activity (**b**); and SOD1, SOD2 and Cat content in the umbilical cord blood plasma of newborns (**c**). Data presented as mean ± SD; *: significance level *p* ≤ 0.05 (p-adjusted) when compared with the control group of the corresponding gestational age.. Ctrl <> 34 are the control groups of the corresponding gestational age. FGR <> 34 are the groups with FGR corresponding to gestational age. AU is an arbitrary unit.

The level of oxidative damage markers (MDA and 4-HNE), the activity of antioxidant enzymes (SOD, CAT, GPx) and the content of SOD1, SOD2 and catalase were assessed in the cord blood plasma of newborns from Cohort I (Figure 2a–c).

A significant increase in the level of the lipid peroxidation products, namely, MDA (17.0 ± 2.2 and 9.5 ± 1.3 nmol/mL, respectively) and a decrease in the GPx activity (23.2 ± 5.4 and 32.7± 2.8 U/mL, respectively) in the cord blood plasma was detected in early-onset FGR compared with late-onset FGR. At the same time, a reduced CAT activity (38.2 ± 9.0 U/mL of protein) was noted based on the background of an increase in its content (76,886 ± 4878 AU), as well as the content of SOD1 (45,644 ± 3148 AU) and SOD2 (68,353 ± 2886 AU) in early-onset FGR. In late-onset FGR, on the contrary, a significant decrease in SOD1 (8122 ± 1218 AU), SOD2 (9226 ± 1145 AU) and Cat (30,729 ± 1924 AU) content was revealed.

**Figure 3:**
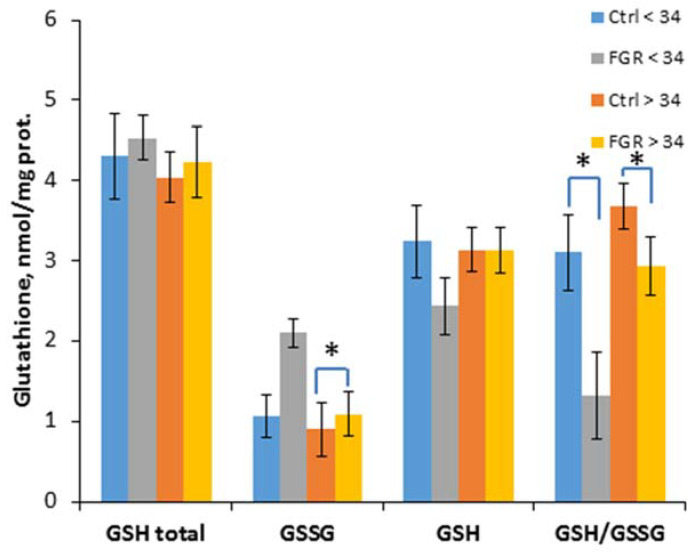
The content of glutathione (total GSH), its reduced (GSH) and oxidized (GSSG) forms, as well as their ratio in the umbilical cord blood plasma of the newborns. Data are presented as mean ± SD; *: significance level *p* ≤ 0.05 (p-adjusted) when compared with the control group of the corresponding gestational age.. Ctrl <> 34 are the control groups of the corresponding gestational age. FGR <> 34 are the groups with FGR corresponding to gestational age.

To evaluate the effectiveness of non-enzymatic antioxidant protection, the content of glutathione and its reduced and oxidized forms was determined, and their ratio was calculated (Figure 3).

A significant decrease of the GSH/ GSSG ratio in early-onset (1.3 ± 0.3 and 3.1 ± 0.5, respectively) and late-onset FGR (2.9 ± 0.4 and 3.7 ± 0.5, respectively) was detected, and an increase of the GSSG content (1.1 ± 0.3 and 0.9 ± 0.2 nmol/mL, respectively) was observed in late-onset FGR.

### 3.2. Correlations of Clinical Assessments, Changes in the Expression of Markers of Pro- and Anti-Oxidant Systems in Newborns with FGR and ROC-Curves

Considering the significant increase in OS markers based on the background of a decrease in the activity of the antioxidant system in newborns with early-onset FGR and a decrease in the level of OS in newborns with late-onset FGR, it seemed interesting to evaluate the relationship of these markers with their clinical assessments using a Spearman’s nonparametric rank correlation (Table 2). It should be noted that the search for correlations was carried out in the total sample of newborns with early-onset and late-onset FGR.

In general, the analysis revealed the relationship between the newborn’s weight and the Apgar scores of their condition at birth with the status of the pro- and anti-oxidant system. In particular, a significant positive correlation was established between the reduced level of GPx and the newborn’s weight (r = 0.6; *p* = 0.03), as well as their Apgar scores at the 1st minute (r = 0.5; *p* = 0.03) and Apgar scores at the 5th minute (r = 0.5; *p* = 0.05). Apgar 1 and Apgar 5 also correlated with a reduced GSH/GSSG (r = 0.6, *p* = 0.01; r = 0.7, *p* = 0.007, respectively), which reflected the weakening of the antioxidant protection. At the same time, a high level of MDA observed in newborns with early-onset FGR, correlated with a low Apgar 1 (r = −0.5; *p* = 0.03). It should be noted that the increase of oxidative stress indicators of SOD1 (r = −0.6; *p* = 0.01), SOD2 (r = −0.6; *p* = 0.02), and the catalase content (r = −0.8; *p* = 0.0005), negatively correlated with the neonatal weight. In addition, the catalase content was associated with a low Apgar 1 (r = −0.6; *p* = 0.03), and SOD2 with Apgar 5 scores (r = −0.7; *p* = 0.003). It is worth pointing out that there were no significant differences in the Apgar 1 and Apgar 5 scores, a newborn’s weight, and the markers of the pro- and anti-oxidant status between the sexes in the study groups.

Taking into account the correlations, it seemed interesting to evaluate the differences in the expression of the above markers depending on the presence or absence of perinatal and neonatal complications in the newborns with early-onset and late-onset FGR (Table 3).

A significant increase in MDA (*p* = 0.04) was revealed in the newborns with ELBW, whereas the CAT activity (*p* = 0.009), tGSH (*p* = 0.01), GSH (*p* = 0.04), and GSH/GSSG ratio (*p* = 0.02) were significantly reduced. At the same time, the SOD1 (*p* = 0.01), SOD2 (*p* = 0.003), and Cat (*p* = 0.004) content was increased. Additionally, in newborns with early FGR and increased echogenicity of the periventricular zones, according to the neurosonography (NSG), the level of the oxidative marker 4-HNE (*p* = 0.03) and the Cat content (*p* = 0.02) were increased.

It is interesting to note that in late FGR in newborns with IP (*p* = 0.04) and hyperbilirubinemia (*p* = 0.02), the 4-HNE expression was reduced, and in newborns with IVH and cerebral cysts, according to NSG data, the SOD1 (*p* = 0.04; *p* = 0.03) and SOD2 contents (*p* = 0.05; *p* = 0.05) were significantly reduced. In addition, an increase in CAT activity (*p* = 0.04) was observed in the presence of cerebral cysts, which confirms the data on a decrease in the OS level in newborns with late-onset FGR based on the background of increased antioxidant protection.

Based on significant expression changes of the pro- and anti-oxidant markers in the cord blood plasma of newborns with FGR, logistic regression models were created with ROC curves to assess the possibility of using the markers in the risk diagnosing of IVH. The model with the combination of MDA + 4-HNE + CAT + GSH (AUC = 1; Se-1; Sp-1; cutoff—0.5) with a high sensitivity and specificity indicates the possibility of predicting the development of IVH immediately after birth.

### 3.3. Comparative Evaluation of miRNAs Expression in Newborn Blood Plasma

A comparative analysis in newborn plasma samples (Cohort II) showed differences in the miR-25-3p and miR-127-3p expression by gestational age (Group I (<32 GA) and Group II (>32 GA)) and neonatal outcomes. The miR-25-3p expression was significantly increased in the newborns with NS (−3.98 (−3.6; −5.32)) relative to TT (−7.91 (−6.01; −9.45)) (*p* ≤ 0.03) only in Group II. There were no statistically significant differences in its expression in newborns from Group I.

**Figure 4:**
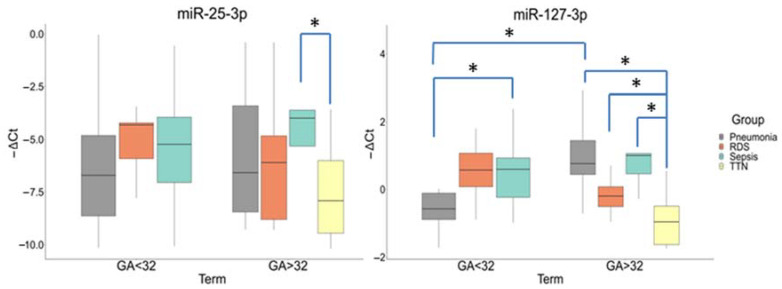
Comparative analysis of the expression for miR-25-3p and miR-127-3p in blood plasma from newborns with perinatal outcomes at before (<32) and after (>32) gestational ages (GA). *: significance level *p* ≤ 0.05 when compared groups with each other. The box diagram shows the medians of −ΔCt values (relative quantification data), the first and third quartiles, and the edges of the statistically significant sample, while the dots denote the emissions.

In Group I, the miR-127-3p expression was significantly increased in newborns with NS (−0.59 (−0.93; 0.23)) relative to IP (0.57 (0.11; 0.89)) (*p* ≤ 0.03). In Group II, its expression was also increased in newborns with NS (1(1.06; 0.46)) (*p* ≤ 0.009), IP (0.76(1.44; 0.44)) (*p* ≤ 0.0001), and RDS (0.2(0.08; 0.51)) (*p* ≤ 0.03) relative to TT. In addition, there were differences in the miR-127-3p expression in newborns with IP between Groups I and II (*p* ≤ 0.004) (Figure 4). 

It is worth pointing out that there were no significant differences in the miRNAs expression between the sexes within the studied groups.

### 3.4. Correlation of Clinical Assessments in Newborns with Perinatal Outcomes and ROC-Curves

To assess the relationship of clinical parameters of newborns (Cohort II), a correlation analysis was performed using the Spearman nonparametric rank correlation method in accordance with the gestational age and the diagnosis at birth. The analysis demonstrated the relationship between the newborns’ weight, assessment of their state according to the scales (i.e., Apgar, Silverman, and NEOMOD) and laboratory parameters in the subgroups. It should be noted that in newborns with IP and RDS, correlations were established only at a period after 32 weeks of gestation (Table 4).

**Figure 5:**
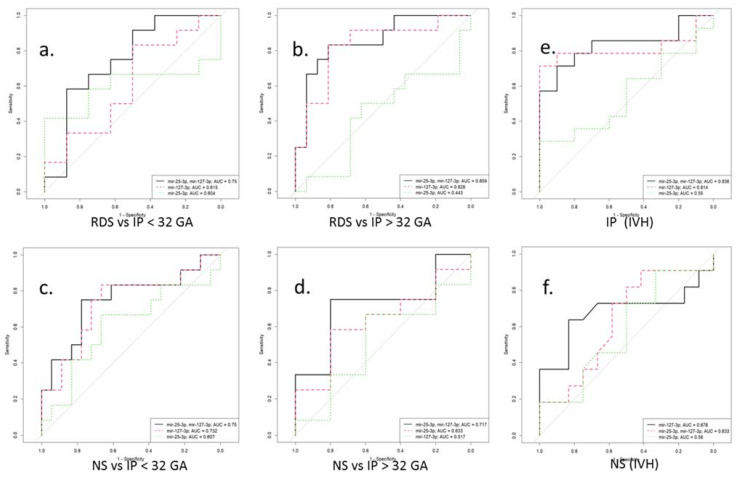
ROC curves for a logistic model for the differential diagnosis of perinatal outcomes (**a**–**d**) by miR-25-3p and miR-127-3p expression in blood plasma of newborns before (<32) and after (>32) gestational age (GA). ROC curves for a logistic model for the risk assessment IVH by miR-25-3p and miR-127-3p expression in blood plasma of newborns with IP (**e**) and NS (**f**) before 32 gestation weeks. RDS is respiratory distress syndrome. IP is intrauterine pneumonia. NS is neonatal sepsis. IVH is intraventricular hemorrhage.

In particular, in newborns with IP, negative correlations were found on the Apgar score at 1 min (r = −0.6, *p* = 0.01) and the Apgar score at 5 min (r = −0.6, *p* = 0.04) with the neutrophil index. The latter correlation was also found in newborns with RDS (r = −0.6, *p* = 0.006), in whom the leukocyte formula also correlated with the Silverman scale (r = 0.5, *p* = 0.04; r = 0.5, *p* = 0.04). In addition, a relationship was established between the newborn’s weight with RDS and the Apgar scores at 5 min (r = 0.7; *p* = 0.04), and the newborn’s weight with NS and the NEOMOD scale (r = −0.9; *p* = 0.04). It is interesting to note that in the newborns with NS, a significant negative correlation was found between an increased miR-127-3p expression and the APGAR 5 score (r = −0.9; *p* = 0.01). Additionally, the indicators of the latter negatively correlated with the Silverman scale (r = −0.8, *p* = 0.04; r = −0.9, *p* = 0.02), and the platelet, with NEOMOD (r = −0.8; *p* = 0.04). At the same time, only in newborns with NS and born before 32 weeks of gestation, were positive correlations of the presence of IVH with thrombocytopenia (r = 0.6; *p* = 0.008) and the NEOMOD scale (r = 0.6; *p* = 0.007) found. The logistic regression models were created with the ROC curves to assess the possibility of using miR-25-3p and miR-127-3p as potential diagnostic markers for the risk development of RDS, IP, and NS in newborns at the corresponding gestational age. Moreover, considering the high incidence of IVH in newborns with IP and NS, born before 32 weeks of gestation, the logistic regression models were created to assess the risk of IVH in these pathologies (Figure 5 and Table 5).

### 3.5. Involvement of miR-25-3p and miR-127-3p in Redox Regulation, Immune and Inflammatory Responses

**Figure 6:**
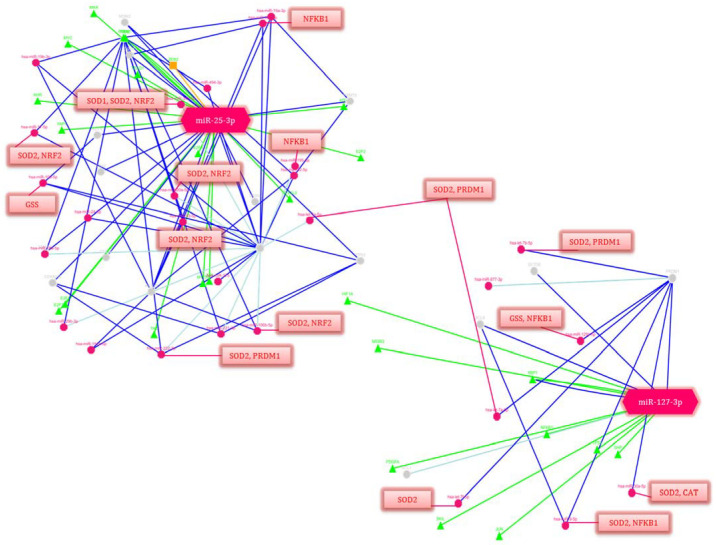
Regulation network of potential gene-targets of miR-25-3p and miR-127-3p (*p*-value ≤ 0.05). Deep blue lines indicate strong evidence (e.g., reporter analysis, Western blot, qRT-PCR or qPCR). The green lines correspond to the regulation of transcription factors. Blue lines are other evidence. Triangles are transcription factors (TF), red circles are miRNAs, gray circles are genes regulated by miRNAs. The red lines indicate the regulation of genes isolated in blocks by these miRNAs. The decoding of the genes’ names is given in Table 6.

Taking into consideration that the correlations between the clinical assessments and changes in the miR-25-3p and miR-127-3p expression in the newborns with perinatal complications, the involvement of these miRNAs in the regulation of signaling pathways and cellular processes was assessed using the miRTarBase 9.0 database, available online: https://mirtarbase.cuhk.edu.cn/ (accessed on 19 December 2022) and GeneCards, available online: https://www.genecards.org/ (accessed on 19 December 2022). (Figure 6).

Among the many pathways and related genes regulated by miR-25-3p and miR-127-3p, both general and specific ones were identified that are involved in the signaling and activation of the immune response: immune homeostasis (in particular, cytokine signaling in the immune system, IL-4 and IL-13 signaling, Class I MHC mediated antigen processing and presentation, the MIF regulation of innate immune cells, immune response CCR3 pathway in eosinophils, leukocyte-intrinsic Hippo pathway functions, immune response IL-1, IL-6, IL-9, IL-17 and IL-23 signaling pathways, the BCR signaling pathway, the Vitamin D receptor pathway, immune response TLR signaling pathways, NFAT in an immune response, and miRNAs involvement in the immune response in sepsis); OS (e.g., the oxidative damage and stress response, FOXO-mediated transcription, and MAPK signaling: oxidative stress, the KEAP1-NFE2L2 pathway, and the regulation of HMOX1 expression and activity); and inflammatory reactions (e.g., NF-kappaB signaling, the CCR5 pathway in macrophages, neuroinflammation and glutamatergic signaling); as well as the response to hypoxia (e.g., cellular response to hypoxia, hypoxic and oxygen homeostasis regulation of HIF-1-alpha and angiogenesis); and apoptosis (e.g., apoptosis and autophagy and the PI3K-Akt signaling pathway) (Table 6).

It should be noted that miR-25-3p and miR-127-3p regulate a significant number of transcription factors that play a key role in the above processes and are the activators of downstream targets. At the same time, a number of miRNAs that are part of the gene network components also regulate the antioxidant response genes (SOD1, SOD2, GSS, and CAT).

## 4. Discussion

It is known that during a physiological pregnancy, in the “mother–placenta–fetus” system, OS increases with the gestational age in parallel with an oxygen consumption by the developing fetus and is accompanied by the compensatory activity of antioxidant enzymes. Morphological and functional disorders of placentation during the early stage of pregnancy, including incomplete vascular remodeling and trophoblast invasion, lead to permanent ischemia/reperfusion in the villous trophoblast zone and OS in the placenta and placental site. With an imbalance in the ROS production and utilization, the observed functional placenta failure is due to a hyper production of ROS, which triggers an uncontrolled cascade of free radical reactions, causing a damaging effect on the mother and fetus [46,47,48]. Within the framework of this paradigm, the functional state of the pro- and anti-oxidant systems of newborns with FGR was assessed.

In order to characterize the severity of the oxidative damage, the concentration of the two main products of lipid peroxidation of cell membranes, namely, MDA and 4-HNE, as well as the activity and content of antioxidant defense enzymes, namely, SOD, CAT and GPx in the umbilical cord blood of the newborns was determined. SODs are the first and most important line of the antioxidant enzymatic defense system against ROS, in particular, superoxide radicals. Depending on their intracellular localization, three isoforms are distinguished, two of which, cytosolic SOD1 (CuZn-SOD) and mitochondrial SOD2 (Mn-SOD) [49], have also been studied in our research. CAT is localized mainly in peroxisomes and catalyzes the breakdown of hydrogen peroxide into water and oxygen [24]. GPx is the main class of enzymes capable of metabolizing hydrogen peroxide and lipid hydroperoxides to the corresponding alcohols in the presence of glutathione, which is converted into its oxidized form [50]. It should be noted that there is an association of elevated OS markers in umbilical cord blood with neonatal outcomes in mothers with PE [51,52] and FGR [53]. In our study, the OS parameters showed significant differences in the MDA based on the background of reduced GPx and CAT activity, as well as an increase in the SOD1, SOD2 and Cat content in early-onset FGR compared to late-onset. The obtained results were quite expected since newborns with a gestation age below 34 weeks are at risk of oxidative damage to the cell membranes in vital tissues and in the blood due to an increased generation of ROS immediately after birth, as well as an ineffective antioxidant defense system [54]. On the contrary, in late-onset FGR, a relatively low OS level remained based on the background of increased activity of antioxidant enzymes, which may indicate a lower risk of oxidative lipid damage in such newborns due to an adequate antioxidant response and better neonatal adaptation compared to newborns with early-onset FGR.

The non-enzymatic system, which is the second line of antioxidant defense, includes glutathione, which plays a significant role in the detoxification of xenobiotics, the neutralization of peroxides and free radicals, as well as in maintaining the intracellular redox status. Glutathione exists in two forms: reduced (GSH) and oxidized (GSSG). In the case of an insufficient level of GSH, a cell is at risk of oxidative damage. Moreover, maintaining an optimal ratio of GSH/GSSG is important for its normal functioning and protection against ROS [55]. Previously, M. Matyas et al. had shown that a decrease in glutathione was directly related to the gestational age and OS in preterm infants [24]. Ahmad et al. also observed a decrease in glutathione in pregnant women with PE [56]. The results of our study are consistent with those of the other authors, demonstrating a significant decrease in the GSH/GSSG ratio, both in early-onset and late-onset FGR. It is interesting to note that, despite the preservation of the first line activity of antioxidant defense in late-onset FGR, the effectiveness of the second line of defense was reduced, which may have been due to the presence of complications in newborns with FGR.

Expecting that changes in the hemodynamics in the placenta with FGR may reflect the relationship of markers of the pro- and anti-oxidant system with the newborn state, we carried out a correlation analysis using the Spearman nonparametric rank method. Significant correlations were found between reduced GPx, GSH, and GSH/GSSG ratios with a low birth weight and Apgar scores. At the same time, a high concentration of MDA was associated with low Apgar scores at 1 min. A significant part of the research demonstrates the relationship between elevated OS markers and a decrease in antioxidants in umbilical cord blood with the risk of developing various neonatal complications, in particular, RDS, sepsis, periventricular leukomalacia, and IVH [27]; thus, the analysis of the above markers can be used to assess prognostic risks, especially for preterm infants. We also found differences in the pro- and anti-oxidant markers’ expression in newborns with growth retardation accompanied by perinatal complications. In particular, in early-onset FGR, in the newborns with ELBW and hyperechogenicity of the periventricular zones (by NSG), the OS markers expression was increased based on the background of the reduced activity of antioxidant enzymes. Conversely, in late-onset FGR, in newborns with congenital pneumonia, hyperbilirubinemia, IVH and cerebral cysts, the opposite picture was observed—a decrease in the OS markers expression based on the background of increased antioxidant protection. It should be noted that the development of perinatal fetal brain damage is one of the most serious consequences of hemodynamic disorders in the placenta vessels associated with OS [57,58]. A number of authors have shown that blood flow resistance in the umbilical artery affects the development of the brain of a newborn in early-onset FGR, and its development in late-onset FGR depends on the dynamics of blood flow in the cerebral artery. Indeed, the presence of a “brain-sparing effect” as a compensatory response to hypoxia does not guarantee the absence of brain tissue damage in a certain period of time [59,60]. In this regard, the logistic regression models were created with ROC curves based on significant changes in the OS markers and antioxidant enzymes expression in the cord blood plasma of the newborns with FGR to assess the risk of IVH from them. The model with the combination of markers of MDA + 4 HNE + CAT + GSH (AUC = 1; cutoff—0.5) indicates the possibility of predicting the development of IVH immediately after birth, which may be important for a preventive therapeutic strategy; however, to confirm the use of this model as a prognostic model, it needs to be verified in a large cohort of newborns with appropriate clinical outcomes.

An important role in maintaining the redox homeostasis of the cell is played by miRNAs—redoximiRs, regulating the expression of ROS generators and components of the antioxidant system [16]. At the same time, ROS can bidirectionally change the expression of the redoximiRs themselves, contributing to the development of a pathological state [61]. It is also significant that OS controls secretion and influences the profile of exosomal miRNAs [14,16], which is important when using the latter as the potential biomarkers of neonatal complications. Our previous study showed a significant change in the redoximiRs expression in the umbilical cord blood of newborns with FGR accompanied by VLBW and RDS. Additionally, a correlation was established with Doppler and clinical parameters of newborns [33]. Based on this data, it seemed interesting to evaluate of miR-25-3p and miR-127-3p expression associated with OS, and immune and inflammatory response in an independent cohort of newborns (before and after 32 weeks of gestation) with different clinical outcomes due to placental dysfunction. The identification of these miRNAs in sepsis, pneumonia, and lung injury, mainly in the adult population, was previously shown by other authors [62,63,64]. A comparative analysis revealed a significant increase in miR-25-3p expression in the plasma of newborns with NS born after 32 weeks of pregnancy. Noteworthy is that a worse prognosis in adult patients with sepsis is associated with a decrease in its expression, since target genes regulated by miR-25-3p are involved in the activation of the NF-κB signaling pathway and the release of inflammatory cytokines by macrophages [62]. Moreover, in a study conducted by Z. Li et al., an association of the rs41274221 miR-25-3p polymorphism with the risk of developing sepsis in newborns with an average gestational age of 32 weeks was shown [65]. It is interesting to note that in the work of L. Yao et al., a decrease in its level in patients with sepsis correlated with an MDA increase and reduced SOD and GPx activity [66]. Meanwhile, in a study performed by Z. Varga et al. [67], the relationship of its reduced expression with a high level of OS was also demonstrated. Considering the data of other authors, we suggest that an increase in the miR-25-3p expression in a cohort of newborns with NS born after 32 weeks of gestation may perform just a protective function that prevents the development of oxidative damage, since our results in a cohort of newborns with late-onset FGR indicated a low OS level on the background of increased antioxidant enzymes activity. Interestingly, the miR-127-3p expression was significantly increased in neonates with NS born before and after 32 weeks of gestation. Moreover, its expression was increased in newborns with IP and RDS born after 32 weeks. MiR-127-3p is considered a molecular switch in macrophage polarization, therefore, its key role in the pathogenesis of diseases associated with inflammation and immune response is assumed. In a model of acute lung injury, for example, it was shown that its increased expression in macrophages led to a significant increase in the production of pro-inflammatory cytokines (M1 polarization) through the activation of a JNK-dependent mechanism, while its inhibition led to the production of anti-inflammatory cytokines (M2 polarization) [64]. In addition, macrophages themselves are able to produce high ROS for activating antimicrobial immune defenses [11]. The data of other authors have demonstrated that miR-127-3p overexpression targeting macrophage CD64 expression, in contrast, inhibits the inflammatory response and reduces lung damage [68]. In the context of our results, such a specific modulation of miR-127-3p expression during the formation of the macrophage phenotype suggests its involvement as an inducer of pro-inflammatory reactions in the genesis of IP, NS, and RDS in newborns.

Despite the revealed significant changes in the studied miRNAs expression in newborns with perinatal complications, only an inverse relationship was established between the increased expression of miR-127-3p and the Apgar score at 5 min in newborns with NS born after 32 weeks of gestation. It should be noted that a low Apgar score is considered as one of the risk factors for the development of sepsis in newborns [69]. In addition, according to Dalili H. et al. low 5 min combined-Apgar scores are associated with an increased risk of neonatal death in all newborns regardless of the gestational age, as well as the risk of IVH and the need for mechanical ventilation [70]. It should be noted that in newborns with NS, born before 32 weeks of gestation, thrombocytopenia was associated with IVH, and IVH with severe dysfunction according to the NEOMOD scale. According to the research of Damaty A. et al. the incidence of thrombocytopenia is significantly higher in preterm infants with IVH. Intriguingly, this may play a decisive role in preventing the development of posthemorrhagic hydrocephalus as a consequence of IVH [71]. The correlation dependences of clinical parameters that were established in newborns with RDS and IP, born after 32 weeks of gestation have also attracted attention. In particular, Apgar scores with neutrophil indices and the Silverman scale with leukocytes and neutrophils, wherein with an increase in the level of the latter, the severity of respiratory problems are elevated. It is known that neutrophils, as integral components of innate immunity, can generate ROS, causing damage to pathogens. On the other hand, they are considered as regulators of adaptive immunity, since they are capable of recruiting and activating T-lymphocytes in inflammation foci [72,73]. It has been previously shown that postnatal neutrophil activation, as a hallmark of systemic inflammation, may contribute to tissue damage with a subsequent increase in pulmonary vascular permeability in preterm infants with RDS [74].

Considering the clinical effects of perinatal complications and the identified correlations based on an miRNA-mediated post-transcriptional gene regulation, it seemed interesting to analyze the involvement of the studied miRNAs in the modulation of the corresponding processes. The signaling pathways and key transcription factors involved in the signaling and activation of the immune response (e.g., PRDM1 and CCL26), immune homeostasis, OS (e.g., NRF2), hypoxia (e.g., HIF1a), and inflammatory responses (e.g., NFKB1) through the activation of downstream targets were retrieved from miRTarBase 9.0 and GeneCards (Table 6). In particular, miR-127-3p is a regulator of PRDM1, a transcription factor that mediates innate and adaptive immune responses. A study by Huang X. et al. showed that PRDM1 plays a significant role in promoting the hyporeactivity of primary T cells, where its overexpression induced an increase in Treg cells and secretion of IL-4, while an inhibition of expression had the opposite effects [75]. It is likely that miR-127-3p, by binding to 3’-UTR of PRDM1, contributes to the inhibition of its expression, leading to the formation of an immune and inflammatory response in newborns with RDS, IP, and NS. Rather intriguing was the involvement of miR-25-3p in both the immune-regulatory and inflammatory signaling pathways, as well as in the regulation of apoptosis, being associated with OS, which has been confirmed by a few studies. In particular, its increased expression during sepsis contributes to the suppression of macrophage migration, as well as a decrease in NFKB1 transcriptional activity and the downstream cytokines TNF-α and IL-6 [62]. Additionally, through the regulation of Nrf2, which plays a leading role in the antioxidant defense system, activating the expression of the corresponding enzymes (e.g., GST, SOD1, and HMOX1) [18], miR-25-3p protects cells from oxidative damage and prevents apoptosis by reducing the ROS and HIF-α, as well as the expression of inflammatory mediators (e.g., IL-1β, TNF-α, IL-6 and MCP-1) [76]. Based on the miR-25-3p-regulated targets and pathways, its increased expression in the neonates with NS that we identified also confirmed its protective function in this cohort of patients.

It is known that exosomal miRNAs, being important mediators of intercellular communication, can act systemically, determining the risk of developing diseases associated with an imbalance of the pro- and anti-oxidant systems. It should also be noted that the differential diagnosis of RDS, IP, and NS is difficult due to the common pathogenetic mechanisms, nonspecific clinical symptoms, similar manifestations, and the absence of specific markers. In this regard, the use of logistic regression analysis made it possible to develop models that indicate the possibility of differentiating these pathologies based on changes in the miR-25-3p and miR-127-3p expression in accordance with gestational age. Moreover, considering the high incidence of IVH in premature newborns, an ROC curve was performed, which demonstrated a high sensitivity (0.79) and specificity (0.9) in assessing the risk of IVH for term newborns with IP based on miR-25-3p and miR-127-3p expression, as well as in preterm infants with NS based on miR-127-3p expression (0.64; 0.83). Interestingly, earlier studies in a cohort of adult patients determined the predictive value of miR-25-3p in sepsis [77] and, likewise, the possibility of using miR-127-3p as a marker for the diagnosis of acute RDS [78]. However, it should be emphasized that further validation studies in large cohorts of newborns are needed to prove the use of the studied miRNAs as biomarkers.

It is worth pointing out that our study had several limitations. First, this was due to the small sample size within each cohort. We paid attention to those in validating the predictive models, which should be performed on large cohorts of newborns with the appropriate clinical outcomes. Second, was the absence of a healthy control group for newborns less than 32 weeks of gestation in the estimation of miRNAs expression. This is due to the fact that premature newborns are at risk of developing various pathologies, primarily due to the immaturity of their organs and systems.

## 5. Conclusions

In our study, we attempted to create a logical chain, the initial link of which was abnormal placentation, leading to a violation of the redox balance. This causes subsequent pathological changes in the fetus, while the final link is the development of neonatal complications, in particular, RDS, pneumonia, sepsis and IVH. At the same time, the relationship between the components of this chain, which are ROS and miRNAs, and coordinating the above processes at the molecular level, have been little studied. In this regard, we made an effort to study the cross violation of ROS and redoximiR in complicated pregnancies. Our study showed a relationship, where the redox balance, and regulation of the immune and inflammatory responses are closely interrelated processes, the modulation of which is mediated by a microRNA expression.

## Figures and Tables

**Table 1:** Clinical characteristic of newborns with adverse outcomes (Cohort II) and maternal diagnoses.

	<32 GA			>32 GA			
	RDS (*n* = 8)	IP (*n* = 12)	NS (*n* = 18)	TT (*n* = 13)	RDS (*n* = 16)	IP (*n* = 12)	NS (*n* = 5)
Gestational age at the time of delivery, weeks	30.6 ± 1.12 *	28.1 ± 1.9	28.06 ± 2.34 ^‡^	35.69 ± 2.56 **	33.73 ± 1.62	35.08 ± 3.03	37.2 ± 2.68 ^‡^
BW, (grams)	1531.7 ± 266.9 *	1077.3 ± 253.4	942.3 ± 302.5 ^‡^	2435.8 ± 357.5	1848.0 ± 308.3 ^‡^	2218.8 ± 705.1	2586.4 ± 680.6
Gender, n (%)							
Male:	3 (37.5)	10 (83.3)	11 (61.2)	6 (46.1)	8 (50)	8 (66.6)	4 (80)
Female:	5 (62.5)	2 (16.7)	7 (38.8)	7 (53.9)	8 (50)	4 (33.4)	1 (20)
APGAR 1 min	6.67 ± 0.5 *	5.17 ± 0.75	4.72 ± 1.23 ^‡^	6.77 ± 1.48	6.93 ± 0.46	5.75 ± 2.63	7.4 ± 0.89
APGAR 5 min	7.44 ± 0.53 *	6.83 ± 0.41	6.61 ± 0.85 ^‡^	7.77 ± 1.17	7.87 ± 0.35	6.92 ± 2.11	8.2 ± 0.84
NEOMOD	2.89 ± 0.6 *	5.17 ± 1.47	5.17 ± 1.54 ^‡^	1.85 ± 0.55 ^&^	2.73 ± 0.7 **	3.58 ± 1.31	4 ± 1.41 ^‡^_,_^Δ^
Silverman	2.56 ± 0.73 *	3.5 ± 0.84	3.22 ± 0.55 ^‡^	1.62 ± 0.96 **	2.67 ± 0.9	1.92 ± 1.56	1.6 ± 1.52
Downes	---	---	---	0.46 ± 0.97	---	1.08 ± 1.62	1.2 ± 1.64 ^‡^
IVH, n (%) Grade (1–2) Grade (3)	1 (12.5) 1 ---	12 (100) 10 2	10 (55.5) 6 4	---	---	2 (16.6) 2 ---	---
WBC (6.0–17.5 × 10^9^ c/L) (absolute count)	8.75 ± 2.83	10.46 ± 4.45	7.76 ± 3.54	17.06 ± 5.46 **	12.38 ± 6.54	16.25 ± 8.75	10.7 ± 6.28
NEUT (1.5–8.5 × 10^9^ c/L) (absolute level) neutropenia (<1500 c/mcl), n (%)	4055 ± 1377 ---	2932 ± 1955 1 (8.3)	2435 ± 2591 ^‡^ 3 (16.6)	8727 ± 4468 ** ---	5916 ± 5120 2 (12.5)	6957 ± 6132 ---	5378 ± 4272 1 (20)
NI (0.17–0.21)	0.06 ± 0.07	0.06 ± 0.06	0.13 ± 0.12	0.05 ± 0.04	0.04 ± 0.05	0.06 ± 0.07	0.05 ± 0.03
Platelet (180–400 × 10^9^ c/L) thrombocytopenia (less 150 × 10^9^ c/L), n (%)	262.4 ± 43.1 *	164.3 ± 61.2	179.7 ± 63.4 ^‡^	291.2 ± 60.9 ^&,Δ^	252.1 ± 61.0	223.3 ± 66.8	195.8 ± 32.7 ^‡^
CRP (less 1.6 mg/l)	1.57 ± 1.45	3.97 ± 7.25	1.9 ± 1.78	1.1 ± 0.75	0.87 ± 0.9	1.51 ± 2.19	5.57 ± 9.1
Maternal diagnoses, n:							
PE	1	1	2	---	---	2	---
FGR	1	4	4	---	---	---	---
PE with FGR	---	---	4	---	---	---	---
GDM	---	1	---	---	---	---	---
Thrombophilia	---	1	5	---	---	---	---
GH	---	3	---	---	---	---	---
AFH	---	---	1	---	---	1	---

BW is birth weight. IVH is an intraventricular hemorrhage. WBC are leucocytes. NEUT are neutrophils. NI is the neutrophil index. CRP is C-reactive protein. GA is gestational age. PE is preeclampsia. FGR is fetal growth restriction. GDM is Gestational Diabetes Mellitus. GH is gestational hypertension. AFH is acute fetal hypoxia. The data are represented in the M ± SD format, where (M) is the mean value and (SD) is standard deviation. *p*-value * is the significance level when comparing RDS and IP; *p*-value ‡ is the significance level when comparing RDS and NS; *p*-value ¶ is the significance level when comparing IP and NS; *p*-value ** is the significance level when comparing TT and RDS; *p*-value Δ is the significance level when comparing TT and NS; *p*-value & is the significance level when comparing TT and IP.

**Table 2:** Correlation of clinical assessments with markers of pro- and anti-oxidant systems in FGR newborns.

	Parameter	r *	*p* **
Weight of newborn	GPx	0.6	0.03
	SOD1	−0.6	0.01
	SOD2	−0.6	0.02
	Cat	−0.8	0.0005
Apgar 1	MDA	−0.5	0.03
	GPx	0.5	0.03
	GSH/GSSG	0.6	0.01
	Cat	−0.6	0.03
Apgar 5	GPx	0.5	0.05
	GSH/GSSG	0.7	0.007
	SOD2	−0.7	0.003

MDA is malondialdehyde. SOD1 and SOD2 are superoxide dismutase. GPx is glutathione peroxidase. GSH is reduced glutathione. GSSG is oxidized glutathione. Cat is the catalase level. r * is a Spearman rank correlation coefficient; *p* ** is the statistical significance of correlation.

**Table 3:** Evaluation of pro- and anti-oxidant markers’ expression depending on the presence or absence of perinatal and neonatal complications in newborns with FGR.

		Absence of Complication	Presence of Complication	*p* *
**<34 GA**				
	**ELBW**			
	MDA	11.4 (10.3; 13.1)	16.7 (16; 17.8)	0.04
	CAT	83.8 (73.95; 88.2)	36.1 (35; 40.4)	0.009
	tGSH	343.2 (247.5; 376.4)	4.6 (4.35; 4.75)	0.01
	GSH	3.27 (3.18; 3.32)	2.6 (2.25; 2.7)	0.04
	GSH/GSSG	2.89 (2.8; 3.31)	1.13 (0.92; 1.64)	0.02
	SOD1	34,576.9 (32,347.1; 35,896.6)	44,368.92 (43,782.74; 46,867.36)	0.01
	SOD2	42,686.2 (41,245.2; 44,501.7)	69,632.2 (65,415.3; 71,929.8)	0.003
	Cat	52,101.5 (50,577.6; 53,371.4)	78,296.4 (73,720.08; 80,756.8)	0.004
	**Hyperechogenicity periventricular zone** **(by neurosonography)**			
	4-HNE	7.15 (6.08; 8.12)	11 (10.85; 11.15)	0.03
	Cat	53,371.4 (51,339.5; 58,266.9)	80,756.8 (79,526.6; 81,987.1)	0.02
**>34 GA**				
	**Intrauterine pneumonia**			
	4-HNE	6.7 (6; 7.9)	5.1 (4.15; 5.5)	0.04
	**Hyperbilirubinemia**			
	4-HNE	6.55 (5.93; 7.78)	4.15 (3.68; 4.62)	0.02
	**IVH**			
	SOD1	29,063.8 (9328.7; 32,210.6)	7648.2 (7373.4; 7978.8)	0.04
	SOD2	36,105.7 (9065.3; 39,472.5)	9903.1 (9113.9; 9943.6)	0.05
	**Cerebral cyst ** **(by neurosonography)**			
	CAT	79.2 (69.2; 86.3)	93.1 (92.9; 95.1)	0.04
	SOD1	29,063.8 (9328.7; 32,210.6)	7098.6 (6973.08; 7373.4)	0.03
	SOD2	36,105.7 (9065.3; 39,472.5)	9903.1 (9113.9; 9993.8)	0.05

MDA is methylmalonic dialdehyde. 4-HNE is 4-hydroxynonenal. SOD1 and SOD2 are superoxide dismutase. tGSH is total glutathione. GSH is reduced glutathione. GSSG is oxidized glutathione. Cat is catalase level. CAT is catalase activity. ELBW is extremely low birth weight. GA is gestational age. The data are represented in the Me (Q1–Q3) format, where (Me) is the median and Q1 and Q3 are the quartiles. *p* * is the statistical significance of correlation.

**Table 4:** Correlation of clinical assessments in newborns with perinatal outcomes.

Respiratory Distress Syndrome			Intrauterine Pneumonia			Neonatal Sepsis		
	r *	*p* **		r *	*p* **		r *	*p* **
APGAR 5—BW	0.7	0.04	APGAR 1—NI	−0.6	0.01	miR-127-3p—APGAR 5	−0.9	0.01
APGAR 5—NI	−0.6	0.006	APGAR 5—NI	−0.6	0.04	NEOMOD—BW	−0.9	0.04
Silverman—NEU	0.5	0.04				APGAR 1—Silverman	−0.8	0.04
Silverman—LEU	0.5	0.04				APGAR 5—Silverman	−0.9	0.02
						Platelet—NEOMOD	−0.8	0.04
						<32 GA		
						Thrombocytopenia—IVH	0.6	0.008
						IVH—NEOMOD	0.6	0.007

BW is the birth weight. NI is the neutrophil index. LEU are leucocytes. NEU are neutrophils. IVH is intraventricular hemorrhage. * r is a Spearman rank correlation coefficient. ** *p* is the statistical significance of correlation.

**Table 5:** Predictive values of miR-25-3p and miR-127-3p in newborns with perinatal outcomes according to gestational age.

miR-25-3p + miR-127-3p	AUC	Sensitivity	Specificity	Cutoff
RDS vs. IP < 32 GA	0.75	0.83	0.75	0.74
RDS vs. IP > 32 GA	0.86	0.83	0.88	0.46
NS vs. IP < 32 GA	0.75	0.75	0.78	0.45
NS vs. IP > 32 GA	0.72	0.75	0.8	0.67
IP (IVH)	0.84	0.79	0.9	0.67
**miR-127-3p**				
NS (IVH)	0.68	0.64	0.83	0.49

**Table 6:** MiRNA-regulated genes, and signaling pathways involved in immune response activation, oxidative stress, inflammatory responses, hypoxia, and apoptosis.

Gene Abbreviation (Ensembl ID)	Gene Name	Description	miRNA * (Reporter Assay, Western Blot, qPCR)	Pathway
**BCL2L11** (ENSG00000153094)	Bcl-2-like protein 11 (BIM)	Belongs to the BCL-2 protein family. The protein encoded by this gene interacts with other members of the BCL-2 protein family and acts as an apoptosis activator. Its expression can be induced by the nerve growth factor (NGF) as well as the transcription factor FKHR-L1, which suggests the role of this gene in the apoptosis of neurons and lymphocytes.	miR-25-3p miR-9-5p miR-106b-5p miR-17-5p miR-32-5p miR-10b-5p miR-181a-5p miR-494-3p miR-375 miR-92a-3p miR-24-3p	FOXO-mediated transcription, Apoptosis and autophagy, MAPK signaling: oxidative stress, Cytokine signaling in immune system, PI3K-Akt signaling pathway.
**CCL26** (ENSG00000006606)	C-C motif chemokine ligand 26	Belongs to the family of cytokine genes involved in immunoregulatory and inflammatory processes.	miR-25-3p	MIF regulation of innate immune cells, CCR5 pathway in macrophages, Immune response CCR3 Pathway in eosinophils.
**NFE2L2 (NRF2)** (ENSG00000116044)	Nuclear Factor, Erythroid 2 Like 2	A transcription factor that plays a key role in the response to oxidative stress. By binding to antioxidant response elements (ARE) present in the promoter region of many cytoprotective genes, it promotes their expression, thereby neutralizing reactive electrophiles. Critical regulator of innate immune response and survival in sepsis by maintaining redox homeostasis and curbing dysregulation of pro-inflammatory signaling pathways. Suppresses the inflammatory response of macrophages by blocking the transcription of pro-inflammatory cytokines and the induction of IL-6.	miR-340-5p miR-17-5p miR-93-5p miR-20a-5p miR-106b-5p	KEAP1-NFE2L2 pathway, Regulation of HMOX1 expression and activity, MAPK signaling: oxidative stress, Class I MHC mediated antigen processing and presentation, Oxidative stress response.
**PRDM1** (ENSG00000057657)	B lymphocyte-induced maturation protein-1	A transcription factor that mediates the innate and adaptive immune response of tissue-resident T-lymphocytes (resident memory T-cells, resident natural killers (TrNK) and natural killers (NKT) in non-lymphoid organs (skin, intestines), as well as the liver and kidneys, which provides immediate immunological protection against reactivating infections or viral reinfection. Controls the maturation of B-lymphocytes in Ig-secreting cells. Acts as a repressor of interferon-beta expression.	miR-127-3p miR-9-5p miR-30a-5p miR-125b-5p miR-222-3p let-7a-5p let-7b-5p let-7f-5p	NF-kappaB signaling, Leukocyte-intrinsic Hippo pathway functions, Vitamin D receptor pathway.
**BCL6** (ENSG00000113916)	B-cell lymphoma 6	Acts as a transcriptional repressor and modulates the transcription of STAT-dependent IL-4 responses in B cells. Necessary for the establishment and maintenance of immunological memory of T and B cells. Suppresses the proliferation of macrophages. It also controls neurogenesis by changing the composition of NOTCH-dependent transcription complexes on selective NOTCH targets, which leads to neuronal differentiation.	miR-127-3p miR-9-5p	Cytokine signaling in Immune system, FOXO-mediated transcription. NF-kappaB signaling, Notch signaling pathways, IL-4 and IL-13 signaling, BCR signaling pathway, IL4-mediated signaling events, Vitamin D receptor pathway.
**HIF1a** (ENSG00000100644)	Hypoxia Inducible Factor 1 Subunit Alpha	A transcription factor that functions as the main regulator of the adaptive cellular response to hypoxia. Activates transcription of more than 40 genes under hypoxic conditions involved in energy metabolism, angiogenesis, apoptosis, protein products which increase oxygen delivery or facilitate metabolic adaptation to hypoxia. Induces the expression of ACE2 and cytokines such as IL1B, TNF, IL6 and interferons in monocytes.	miR-20a-5p miR-18a-5p miR-17-5p let-7b-5p miR-93-5p miR-106b-5p miR-494-3p	Cellular response to hypoxia, Hypoxic and oxygen homeostasis regulation of HIF-1-alpha, Cytokine signaling in Immune system, Class I MHC mediated antigen processing and presentation, IL-4 and IL-13 signaling, Angiogenesis, Notch and PI3K-Akt signaling pathways, Vitamin D receptor pathway.
**NFKB1** (ENSG00000109320)	Nuclear Factor Kappa B Subunit 1	A transcriptional regulator that is activated by various intracellular and extracellular stimuli such as cytokines, free radicals, ultraviolet radiation, bacterial or viral products. Inadequate activation of NFKB is associated with a number of inflammatory diseases, while persistent inhibition of NFKB leads to abnormal development of immune cells or retardation cell growth. NFKB is a critical regulator of the immediate early response to viral infection.	miR-9-5p miR-92a-3p miR-195-5p let-7a-5p	Cytokine signaling in Immune system, Immune response IL-1, IL-6, IL-9, IL-17 and IL-23 signaling pathway, Immune response TLR signaling pathways, NFAT in immune response, CCR5 pathway in macrophages, Notch, MAPK and PI3K-Akt signaling pathways, Class I MHC mediated antigen processing and presentation, miRNAs involvement in the immune response in sepsis, Neuroinflammation and glutamatergic signaling, Oxidative damage and stress response.

Note. * These validations are based on the miRTarBase information.

## Data Availability

The datasets used and/or analyzed in the current study are available from the corresponding author on reasonable request.

## Supplementary Materials

The following supporting information can be downloaded at: https://www.mdpi.com/article/10.3390/antiox12010006/s1. Table S1: Clinical characteristic of newborns with adverse outcomes (Cohort II).
