# Supplementary material for: Interrelation between miRNAs Expression Associated with Redox State Fluctuations, Immune and Inflammatory Response Activation, and Neonatal Outcomes in Complicated Pregnancy, Accompanied by Placental Insufficiency
Source: Antioxidants (Basel). 2022 Dec 21;12(1):6. doi: 10.3390/antiox12010006 (PMC9854567; doi:10.3390/antiox12010006)
Supplement: Supplementary file 1 [file antioxidants-12-00006-s001.zip › antioxidants-1990780-supplementary.pdf]

Table S1. Clinical characteristic of newborns with adverse outcomes (II cohort)

	**< 32 GA**			**> 32 GA**			
	**RDS (n=8)**	**IP (n=12)**	**NS (n=18)**	**TT (n=13)**	**RDS (n=16)**	**IP (n=12)**	**NS (n=5)**
DIC, n (%)	0	7 (58.3)	10 (55.5)	0	0	4 (33.3)	1 (20.0)
Neonatal hyperbilirubinemia, n (%)	6 (75.0)	7 (58.3)	10 (55.5)	0	8 (50.0)	5 (42.0)	0
Anaemia, n (%)	3 (37.5)	2 (16.6)	6 (33.3)	0	1 (6.2)	1 (8.3)	0
Neonatal asphyxia, n (%)	0	6 (50.0)	14 (77.7)	3 (23.0)	0	3 (25.0)	0
Cerebral ischemia, n (%)	0	4 (33.3)	3 (16.6)	0	0	2 (16.6)	0
Neonatal seizures, n (%)	0	3 (25.0)	6 (33.3)	0	0	3 (25.0)	1 (20.0)
Gastrointestinal dyskinesia, n (%)	3 (37.5)	1 (8.3)	4 (22.2)	0	1 (6.2)	0	0
Gastric hemorrhage, n (%)	0	2 (16.6)	6 (33.3)	0	0	1 (8.3)	1 (20.0)
NEC, n (%)	0	2 (16.6)	3 (16.6)	0	0	0	0

DIC is a disseminated intravascular coagulation. NEC is nonspecific enterocolitis. GA is a gestational age. RDS is a respiratory distress syndrome. IP is an intrauterine pneumonia. NS is a neonatal sepsis. TT is a transient tachypnea.
